# Regional patterns of genetic variants in expanded carrier screening: a next-generation sequencing pilot study in Fujian Province, China

**DOI:** 10.3389/fgene.2025.1527228

**Published:** 2025-05-12

**Authors:** Danhua Guo, Nani Zhou, Qianqian He, Na Lin, Shuqiong He, Deqin He, Yifang Dai, Ying Li, Xuemei Chen, Hailong Huang, Jia Jia, Hua Cao, Liangpu Xu

**Affiliations:** ^1^ Department of Medical Genetic Diagnosis and Therapy Center, Fujian Maternity and Child Health Hospital, College of Clinical Medicine for Obstetrics & Gynecology and Pediatrics, Fujian Medical University, Fuzhou, China; ^2^ Fujian Provincial Key Laboratory of Prenatal Diagnosis and Birth Defect, Fuzhou, China; ^3^ Shanghai Fujungenetics Biotechnology Co., Ltd., Shanghai, China; ^4^ Fujian Key Laboratory of Women and Children’s Critical Diseases Research, Fujian Maternity and Child Health Hospital, Fuzhou, China

**Keywords:** expanded carrier screening, gene, genetic disease, next-generation sequencing, variant

## Abstract

**Background:**

This pilot study aimed to characterize the regional distribution of genetic variants associated with autosomal recessive and X-linked recessive (AR/XLR) conditions in Fujian Province, Southeast China, to inform the development of targeted carrier screening programs.

**Methods:**

An expanded carrier screening (ECS) panel utilizing next-generation sequencing (NGS) technology was designed to analyze 332 genes associated with 343 AR/XLR conditions. The panel was applied to 440 samples obtained from individuals in Fujian Province. Single nucleotide variants and copy number variations (CNVs) were identified and analyzed using a multidimensional approach that incorporated demographic characteristics, carrier frequencies, and the genetic burden of AR/XLR diseases.

**Results:**

A total of 511 variants were detected among the 440 participants, including 43 CNVs (8.41%), affecting 133 genes associated with 123 conditions. The mean number of pathogenic or likely pathogenic variants per sample was 1.16. The highest genetic burden was observed in couples seeking medically assisted reproduction (MAR group), who had histories of fetal loss, second- or third-trimester abnormalities, or postnatal abnormalities. In clinical settings, the percentage of at-risk couples (ARCs) was 6.36% (n = 14), involving seven conditions, with no statistically significant difference in ARC incidence between couples undergoing genetic screening (GS group) and the MAR group. The cumulative carrier rate for 28 genes was ≥1/100. Recurrent variants in *GAA*, *GALT*, *CYP1B1*, and *MEFV* were identified, exhibiting distinct regional patterns compared to previously reported variants in the Han Chinese population.

**Conclusion:**

NGS-based ECS demonstrates significant potential for assessing the genetic burden of AR/XLR conditions and identifying ARCs in Fujian Province. However, before integrating ECS into regional public health initiatives, the development of a region-specific, curated disease panel is necessary to optimize screening efficacy and clinical utility.

## 1 Introduction

Globally, an estimated 5,000–8,000 monogenic diseases follow Mendelian inheritance patterns, with more than 2,000 exhibiting autosomal recessive (AR) inheritance (Prakash et al., 2016; Xiao and Lauschke, 2021). Genetic disorders are present in approximately 2%–3% of live births and contribute to approximately 20% of infant mortality and 10% of infant hospital admissions, collectively affecting over 350 million individuals worldwide (Baird et al., 1988; Kumar et al., 2001).

Carrier screening has been widely implemented as an effective strategy for reducing the incidence of single-gene recessive disorders. For example, a study conducted by Marinakis et al. in Europe, utilizing an expanded carrier screening (ECS) panel targeting 176 genes, reported that 47.50% of participants carried pathogenic variants, with 1.67% of couples identified as at risk of having offspring affected by at least one AR condition (Marinakis et al., 2024). Similarly, an ECS analysis by Strauss et al. in New York City identified a carrier rate of 71.85% and the percentage of ARC was 9.46% (Strauss et al., 2023). In 2024, Li et al. conducted a study involving 3,024 individuals from South and Southwest China using a 220-gene ECS panel, identifying 1,885 individuals with pathogenic or likely pathogenic variants (PLPVs) and 128 AR carrier couples, with a theoretical offspring incidence rate of 2.12% (Huang et al., 2024).

In 2024, China released three expert consensus statements on carrier screening, outlining recommendations regarding target populations, disease inclusion criteria, and screening strategies tailored to the national healthcare landscape. While these recommendations align with the general principles established by the American College of Medical Genetics and Genomics (ACMG) and the American College of Obstetricians and Gynecologists (ACOG), specific modifications were proposed. For instance, given the significant disparities in economic development, healthcare infrastructure, and education levels across different regions in China, a standardized national carrier screening program encompassing a large number of conditions is not recommended. Instead, a regionally tailored approach is advised, allowing screening programs to be adapted based on the capabilities of local medical institutions and the prevalence of specific conditions (Liu, 2024; Huang and Xu, 2024; Lu et al., 2024).

The prevalence of pathogenic variant carriers for monogenic conditions in the general population is increasingly recognized. Individuals with a single recessive pathogenic or likely pathogenic variant allele typically remain asymptomatic. Carrier screening, along with prenatal screening for Down syndrome and chromosomal aneuploidies, serves as a critical tool for birth defect prevention. In China, limited awareness and insufficient proficiency among healthcare professionals in interpreting genetic variations pose significant barriers to the integration of genetic testing into routine clinical practice (An et al., 2024). Consequently, many recessive genetic conditions remain undetected in standard prenatal care, highlighting the importance of early carrier identification to enhance congenital disorder prevention efforts.

Next-generation sequencing (NGS)-based ECS enables the simultaneous detection of multiple gene variants associated with AR and X-linked recessive (XLR) conditions in phenotypically unaffected individuals, either before conception or during early pregnancy (Henneman et al., 2016). In addition to facilitating reproductive decision-making, ECS has the potential to contribute to national birth defect prevention initiatives by serving as a robust tool for the primary prevention of recessive genetic disorders (Hout et al., 2018). ECS provides couples with comprehensive reproductive options based on their genetic risk profiles, including natural conception, prenatal diagnosis, preimplantation genetic testing, gamete donation, and early planning or interventions for potentially affected offspring (Hout et al., 2018).

International organizations and professional societies have established clear guidelines to support the clinical implementation of ECS, defining standardized criteria for disease selection, testing parameters, and genetic counseling protocols (Edwards et al., 2015; Wilson et al., 2016; Author anonymous, 2017a; Author anonymous, 2017b). This framework facilitates the integration of ECS into routine clinical practice, potentially reducing the risk of adverse pregnancy outcomes (Kingsmore, 2012).

In China, current targeted carrier screening initiatives primarily focus on specific conditions such as hereditary hearing impairment, spinal muscular atrophy (SMA), and thalassemia (Yin et al., 2013; Hu et al., 2021; Li et al., 2020; Zhang et al., 2020; Jiang et al., 2017; Lai et al., 2017). However, the application of NGS-based ECS remains largely restricted to research settings and clinical trials. Several challenges impede its widespread clinical adoption. First, although ACMG and ACOG guidelines advocate for the inclusion of population-specific conditions in ECS, genetic heterogeneity within the Chinese population and the lack of region-specific disease prevalence data complicate the standardization of screening protocols. Second, standard bioinformatics approaches often encounter difficulties in differentiating high-homology genomic regions (e.g., *SMN1*, *CYP21A2*, *HBA1/HBA2*) or accurately detecting copy number variations (CNVs) (e.g., *DMD*) using short-read NGS technology, despite these genes being commonly included in newborn and carrier screening programs and frequently associated with congenital disorders in Southeastern China (Li et al., 2021; Zhao et al., 2021; Wang et al., 2023). To overcome these limitations, additional genetic testing techniques such as multiplex ligation-dependent probe amplification (MLPA) and Sanger sequencing with nested long-fragment polymerase chain reaction (PCR) are often employed. However, these methods increase the overall workload and are less feasible for high-throughput screening. Therefore, advancements in NGS technology, particularly in CNV detection and the analysis of highly homologous regions, are required. Improvements in probe design and bioinformatics pipelines will be essential for enhancing the accuracy and clinical applicability of ECS.

In this study, an integrated ECS approach was developed, combining NGS with advanced bioinformatics methodologies to analyze 332 genes for single nucleotide variants (SNVs), insertions/deletions (indels), and exonic CNVs within a single panel. To assess its efficacy, 220 couples attending a medical genetics diagnosis and treatment center were recruited. By characterizing the PLPV carrier profiles within this cohort, this study aimed to delineate the genetic variant spectrum of common autosomal recessive conditions, evaluate the genetic burden, and identify prevalent AR monogenic birth defects in Southeastern China.

## 2 Materials and methods

### 2.1 Ethical compliance

Between August 2021 and September 2022, a total of 220 couples (440 individuals) attending the center were enrolled in this study. Participants were categorized into two groups based on the indication for genetic evaluation: the genetic screening (GS) group and the medically assisted reproduction (MAR) group. The MAR group was further sub-divided into three subgroups based on clinical presentation: infertility (MAR-group A), spontaneous abortion (MAR-group B), and abnormalities occurring during the second or third trimester or postnatally (MAR-group C). All participants provided written informed consent for the use of peripheral blood samples in clinical research.

Peripheral blood sample collection was conducted in accordance with the ethical principles outlined in the Declaration of Helsinki. The study protocol and utilization of clinical data were reviewed and approved by the Ethics Committee of Fujian Provincial Maternity and Children’s Hospital (Ethical Approval Number: 2021KP031).

### 2.2 Research design and ECS panel

In this study, 220 couples were prospectively evaluated for AR and XLR single-gene disorders. A total of 440 participants with high-quality DNA samples were included, all of whom sought medical services related to pregnancy management or MAR. The ECS panel was designed to screen for 343 single-gene AR/XLR conditions, targeting 332 genes (Supplementary Table S1). Detailed criteria for disease selection and targeted genomic regions are provided in the Supplementary Methods.

### 2.3 Genetic testing methods and variant interpretation

**FIGURE 1:**
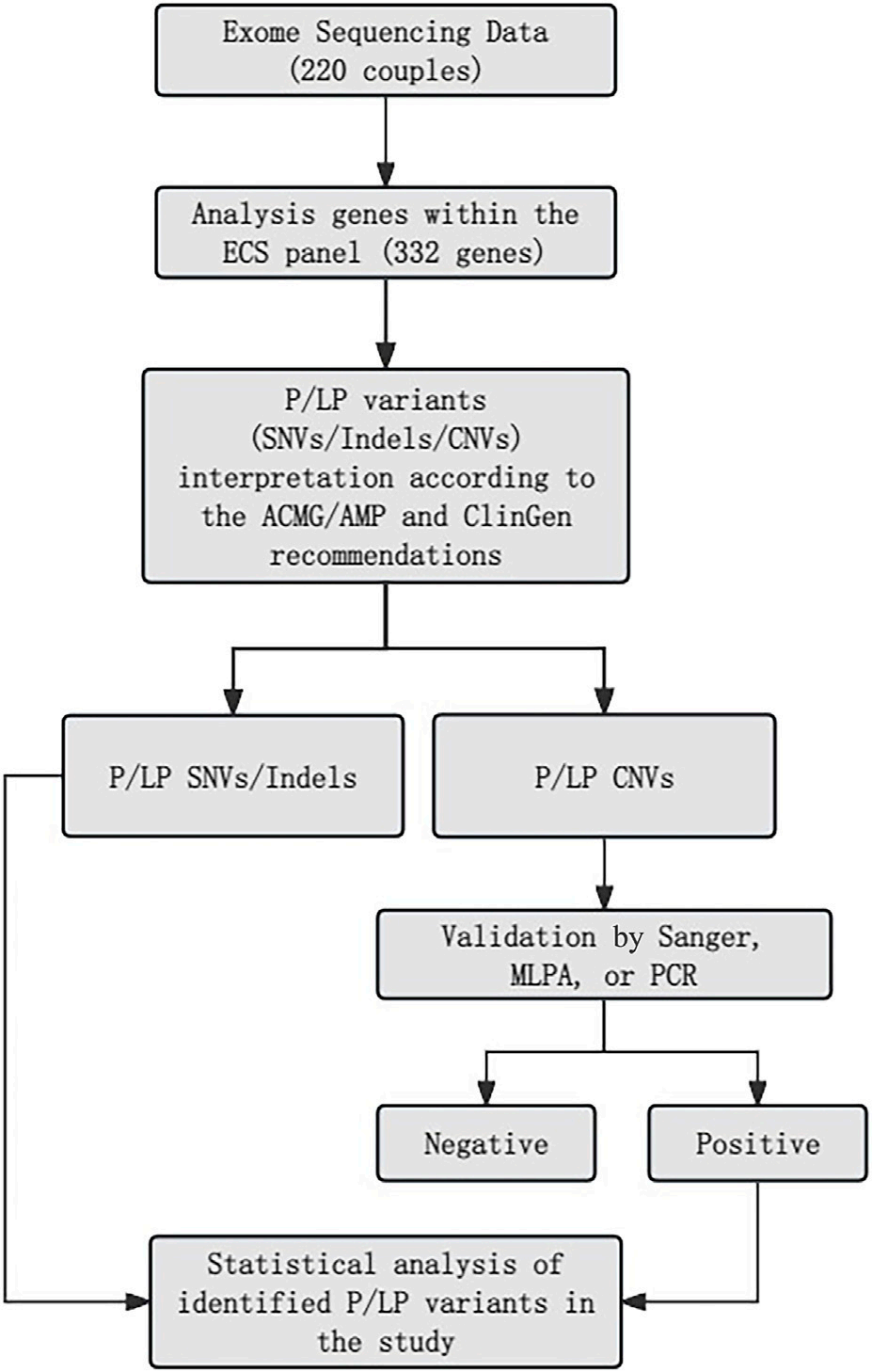
Main filters applied for variant prioritization.

All samples underwent analysis using an NGS-based ECS panel (Figure 1). Variants, including SNVs, indels, and exon-level CNVs, were identified. Variant interpretation was performed following the guidelines established by the ACMG and the Association for Molecular Pathology (Richards et al., 2015; Strom et al., 2021). To validate PLPVs located in highly homologous regions and confirm the presence or absence of specific exons, additional methods such as Sanger sequencing, MLPA, and quantitative PCR (qPCR) were employed (Chen et al., 2023). A comprehensive description of the testing methods is provided in the Supplementary Methods.

### 2.4 Bio-informatics and statistical analysis

Data processing, analysis, and visualization were performed using custom Python and R scripts. ARCs were identified based on the presence of PLPVs in the same gene within a couple or the identification of X-linked PLPVs in female participants (Xi et al., 2020). The cumulative carrier rate (CCR) was calculated using the methodology outlined by Guo and Gregg (2019). Continuous data with a normal distribution were compared between groups using a two-sided t-test, while categorical data were analyzed using the chi-squared test. A *p*-value <0.05 was considered statistically significant. Disease incidence was estimated using the Hardy-Weinberg equilibrium formula based on CCR. Functional annotation of the selected gene list was performed using the DAVID web server. (Sherman et al., 2022).

## 3 Results

### 3.1 Demographic analysis

Among the 440 participants, 84.77% (n = 373) identified as Han Chinese, while 2.73% (n = 12) belonged to one of four ethnic minority groups in China (Manchu, Miao, She, and Zhuang). Ethnicity was not reported for 12.50% (n = 55) of participants. The mean age of female participants was 31.30 years (range: 21–44 years), and the mean age of male participants was 32.89 years (range: 23–57 years) (Table 1; Supplementary Table S2).

**TABLE 1:** Population demographics and classification by screening indication.

Research category		Sex	Average age (Year)	Total no. Of samples	No. Of samples carrying p/lp variant	Total no. Of variants	Carrier rate (%)	MBP (mean variant burden per sample)	X^2^, *p*-value
Total sample analysis		Female	31.30	220	154	260	70.00%	1.18	X^2^ = 0.17, *p* = 0.68
		Male	32.89	220	150	251	68.18%	1.14	
Ethnicity	Han Chinese	Female	31.38	186	128	210	69.00%	1.13	Female^g^: X^2^ = 1.29, *p* = 0.56
		Male	32.88	187	127	214	68.00%	1.14	
	Chinese ethnic minorities^a^	Female	31.29	7	6	14	86.00%	2.00	Male^h^: X^2^ = 0.29, *p* = 0.86
		Male	33.40	5	3	6	60.00%	1.20	
	Other^b^	Female	30.81	27	20	36	74.00%	1.33	
		Male	32.85	28	20	31	71.00%	1.11	
Screening indication	GS-Group^c^	Female	30.30	79	50	87	63.00%	1.10	Female^i^: X^2^ = 2.64, *p* = 0.10
		Male	32.23	79	52	89	66.00%	1.13	
	MAR-group A^d^	Female	33.70	20	15	25	75.00%	1.25	Male^j^: X^2^ = 0.32, *p* = 0.57
		Male	34.30	20	16	25	80.00%	1.25	
	MAR-group B^e^	Female	31.52	94	68	113	72.00%	1.20	
		Male	33.13	94	63	103	67.00%	1.10	
	MAR-group C^f^	Female	31.48	27	21	35	78.00%	1.30	
		Male	32.89	27	19	34	70.00%	1.26	

Note:

^a^ : Chinese Ethnic Minorities include the Manchu, Miao, She, and Zhuang ethnic groups.

^b^ : Refers to individuals with unclear or unknown ethnic information.

^c^ : GS-Group: Genetic screening group.

^d^ : MAR-group A: Infertility group.

^e^ : MAR-group B: Spontaneous abortion group.

^f^ : MAR-group C: Second and third trimesters or postnatal abnormalities group.

^g^ : The *p*-value for carrier rates among females across the Han, ethnic minorities, and “Other” groups, calculated using the chi-square test.

^h^ : The *p*-value for carrier rates among males across the Han, ethnic minorities, and “Other” groups, calculated using the chi-square test.

^i^ : The *p*-value for carrier rates among females in the GS group vs MAR groups (A, B, C), calculated using the chi-square test.

^j^ : The *p*-value for carrier rates among males in the GS group vs MAR groups (A, B, C), calculated using the chi-square test.

### 3.2 Carrier rate and genetic burden per sample

**FIGURE 2:**
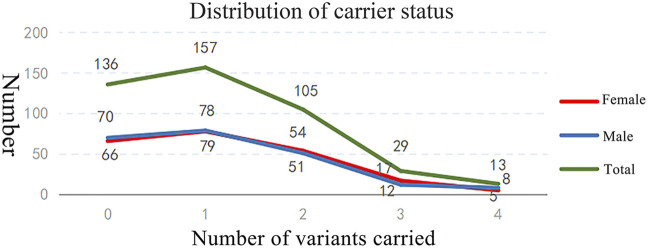
Distribution of carriers with identified genetic variants.

Of the 440 individuals screened, 304 (69.09%) were identified as carriers of at least one AR or XLR condition, comprising 154 females (carrier rate: 70.00%) and 150 males (carrier rate: 68.18%) (Table 1). A total of 511 PLPVs were identified across 133 genes (Supplementary Table S3). Among carriers, 51.64% harbored one variant, 34.54% harbored two variants, 9.54% harbored three variants, and 4.28% harbored four variants. The distribution of variants was comparable between male and female carriers (Figure 2).

The overall genetic burden per sample was 1.16. Among the 220 female participants, 260 variants were detected, yielding a genetic burden of 1.18. Among these, eight were X-linked variants, corresponding to an X-linked recurrent variant rate of 3.63% (8/220). In the 220 male participants, 251 variants were identified, with a genetic burden of 1.14.

Further subgroup analysis examined carrier rates by sex, ethnicity, and testing indication. No statistically significant differences were observed in carrier rates among ethnic groups by gender (*p* > 0.05).

Among females, the carrier rate was 63.29% in the GS group and 73.76% in the MAR group, with no statistically significant difference (χ^2^ = 2.642, *p* = 0.104). Among males, the carrier rate was 65.82% in the GS group and 69.50% in the MAR group, also showing no significant difference (χ^2^ = 0.316, *p* = 0.574).

In MAR-group A, the male carrier rate exceeded the female carrier rate, representing the highest observed carrier frequency among all subgroups. However, the genetic burden was comparable for both sexes at 1.25. In MAR-group B and MAR-group C, females exhibited higher carrier rates than males, though the differences were not statistically significant (MAR-group B: χ^2^ = 0.629, *p* = 0.526; MAR-group C: χ^2^ = 0.386, *p* = 0.757).

### 3.3 Allele frequencies and cumulative carrier rates of identified genes and diseases

Allele frequencies (AFs) and CCR for each identified gene and associated condition were calculated based on the detected variants. These findings were compared to data from previous ECS studies in Chinese populations and previously reported recurrent PLPVs in Chinese individuals. A total of 28 genes with a CCR of ≥1/100 were identified, spanning 8 disease categories (Table 2). Several of these conditions exhibited higher carrier rates than those documented in the literature.

**TABLE 2:** Cumulative carrier rate (CCR) and allele frequencies of the top23 diseases.

Disease system	Disease	Gene	Number	CCR	This study (1 in -)	CCR without low-penetrance variants (1 in -)	References (1 in -)	References (PubMed ID)
Auditory	Non-syndromic hearing loss, *GJB2*-related	*GJB2*	88	0.19	5	44	27	32,573,981
Hematological	Thalassemia	*HBA1/HBA2*	25	0.07	14	—	13	35,945,425
		*HBB*	10					
Metabolic/Endocrine	Crigler-Najjar Syndrome	*UGT1A1*	17	0.04	38	444	61 in Amish	GeneReviews
Auditory	Pendred Syndrome	*SLC26A4*	15	0.03	33	—	47	32,573,981
Metabolic/Endocrine	Phenylalanine Hydroxylase Deficiency Disease	*PAH*	15	0.03	33	—	53	32,573,981
Metabolic/Endocrine	Wilson Disease	*ATP7B*	15	0.03	33	—	43	32,573,981
Auditory	Usher Syndrome	*USH2A*	12	0.03	35	—	37	34,285,390
		*CLRN1*	1					33,422,976
Nervous	Krabbe Disease	*GALC*	12	0.03	34	—	37	32,573,981
Hematological	Hfe-Associated Hemochromatosis	*HFE*	10	0.02	44	444	11	26,633,544
Ophthalmologic	Retinitis Pigmentosa	*EYS*	6	0.02	49	—	—	—
		*CERKL*	2					
		*FAM161A*	1					
Metabolic/Endocrine	Citrin Deficiency	*SLC25A13*	9	0.02	50	—	26	32,573,981
Metabolic/Endocrine	Congenital Adrenal Hyperplasia Due to 21 Hydroxylase Deficiency	*CYP21A2*	9	0.02	50	—	21	34,285,390
Nervous	Tricho-Hepato-Enteric Syndrome	*SKIC3*	8	0.02	55	—	—	—
Musculoskeletal	Spinal Muscular Atrophy	*SMN1*	8	0.02	55	—	65	32,573,981
Metabolic/Endocrine	Glucose-6-Phosphate Dehydrogenase Deficiency	*G6PD*	8	0.02	55	—	48	33,051,526
Metabolic/Endocrine	Primary Carnitine Deficiency	*SLC22A5*	8	0.02	63	—	71	32,573,981
Metabolic/Endocrine	Galactosemia	*GALT*	7	0.02	63	—	110	34,285,390
Nervous	Progressive extraocular muscle paralysis	*POLG*	7	0.02	63	—	188	34,285,390
Metabolic/Endocrine	Pompe disease	*GAA*	7	0.02	63	—	74	34,285,390
Renal	Gitelman syndrome	*SLC12A3*	5	0.01	88	—	50	35,894,287
Immune	Familial Mediterranean fever	*MEFV*	5	0.01	88	—	20 in Hungary	26,399,837
Metabolic/Endocrine	Cystic fibrosis	*CFTR*	5	0.01	88	—	24	34,285,390
Ophthalmologic	Primary congenital glaucoma	*CYP1B1*	5	0.01	88	—	50	34,536,459

Within the metabolic/endocrine anomalies category, ten genes demonstrated an allele frequency (AF) of ≥1/200. The highest-frequency variants were predominantly observed in the auditory anomaly category, with *GJB2* exhibiting the highest carrier rate of 20.0% (1/5). However, after excluding the low-penetrance *GJB2* variant c.109G > A (p.Val37Ile), the adjusted carrier rate decreased to 1/44.

In this cohort, the prevalence of thalassemia, caused by pathogenic variants in *HBA1*/*HBA2* and *HBB*, was 1/13, which aligns with previously reported findings. A total of 12 conditions exhibited a carrier frequency exceeding 1/50. However, some conditions, including Crigler-Najjar syndrome (*UGT1A1*) and HFE-related hemochromatosis (*HFE*), were associated with high-frequency, low-penetrance variants, specifically c.1091C > T (p.Pro364Leu) in *UGT1A1* and c.187C > G (p.His63Asp) in *HFE*. When these low-penetrance variants were excluded, the carrier rate was reduced to 1/444.

Four disease categories—dermatological, respiratory, hepatic, and multisystem diseases—did not include genes with high variant frequencies.

Several recurrent PLPVs were identified in genes previously reported in the literature. Notably, the variant spectra of genes such as *GAA* and *GALT* (associated with metabolic/endocrine anomalies), *MEFV* (associated with immune anomalies), and *CYP1B1* (associated with nervous system anomalies) exhibited differences compared to previously published data on Chinese populations (Supplementary Table S4).

### 3.4 Types of detected variants

A total of 468 (91.59%) SNVs/indels and 43 (8.41%) CNV exons were detected using the 332-gene ECS panel (Supplementary Tables S5, S6). The majority of SNVs/indels (n = 453, 88.65%) were heterozygous, while five couples were identified as homozygous carriers for SNVs/indels in the genes *GJB2*, *UGT1A1*, *G6PD*, and *SLC22A5* (Supplementary Table S7). None of these individuals reported phenotypic manifestations consistent with these conditions.

Among the 43 cases with CNV exons, 24 were female and 19 were male, representing 9.23% and 7.57% of the total CNV variations within each respective group. Alpha-thalassemia was identified as the most prevalent CNV-associated condition, with CNV variants αα/--^SEA^, αα/-α^3.7^, and αα/-α^4.2^ detected in 23 cases (53.49%) (Table 3).

**TABLE 3:** Genotype-phenotype correlation of CNVs.

Gene	Sex	Variants	Disease	Number of samples	Total number of samples
*HBA1/HBA2*	Female	αα/-^-SEA^	Alpha-Thalassemia	8	23
		αα/-α^3.7^		4	
		αα/-α^4.2^		1	
	Male	αα/--^SEA^		6	
		αα/-α^3.7^		3	
		αα/-α^4.2^		1	
*SMN1*	Female	ex.7_8del (p.?)	Spinal Muscular Atrophy	5	8
	Male	ex.7_8del (p.?)		3	
*GLDC*	Female	ex.2del (p.?)	Glycine encephalopathy	1	2
	Male	ex.1-8del		1	
*NPHP1*	Female	ex.1_20del (p.?)	Joubert syndrome	2	2
*CYP21A2*	Male	ex.1_10del (p.?)	Congenital Adrenal Hyperplasia Due to 21 Hydroxylase Deficiency	1	1
*DCLRE1C*	Male	ex.1_3del (p.?)	Severe Combined Immunodeficiency with Sensitivity to Ionizing Radiation	1	1
*FAM161A*	Male	ex.4-5del (p.?)	Retinitis Pigmentosa	1	1
*FANCA*	Female	ex.7-14del (p.?)	Fanconi Anemia	1	1
*PMM2*	Female	ex.2del p.?	Congenital Disorder of Glycosylation, Type Ia	1	1
*SACS*	Male	ex.1_8del (p.?)	Spastic Ataxia, Charlevoix-Saguenay Type	1	1
*SGCG*	Male	ex.5_10del (p.?)	Autosomal Recessive Limb-Girdle Muscular Dystrophy Type 2c	1	1
*VPS13B*	Female	ex.17del (p.?)	Cohen Syndrome	1	1

Eight cases exhibited CNVs in the *SMN1* gene, all involving a deletion of exons 7–8, which is pathogenic for SMA, irrespective of sex. Additionally, rare CNV exons were identified in genes *DCLRE1C*, *FAM161A*, *PMM2*, *SACS*, *SGCG*, and *VPS13B*, with annotations available in the ClinVar and HGMD databases.

### 3.5 Characteristics of ARCs

Among the 220 tested couples, 6.36% (n = 14) were identified as ARCs, carrying pathogenic or likely pathogenic (P/LP) variants associated with seven distinct conditions (Supplementary Table S8). Six couples were identified as carriers of P/LP variants in the same gene associated with AR conditions, conferring a 25% (1 in 4) risk of having offspring affected by the respective disorder. The conditions identified included metachromatic leukodystrophy, SMA, Krabbe disease, phenylalanine hydroxylase (PAH) deficiency, Pendred syndrome, and alpha-thalassemia.

Among these couples, Couples 12, 55, and 185 belonged to MAR-group C. Couple 12 had a child diagnosed with metachromatic leukodystrophy, with genetic testing confirming that both parents carried P variants in the *ARSA* gene. Couple 55 experienced the loss of their infant at 3 months of age due to respiratory failure and muscle weakness. Further genetic analysis revealed that both parents carried a deletion of exons 7 and 8 in the *SMN1* gene, confirming a diagnosis of SMA. Couple 185 had a child with hearing impairment, with genetic testing identifying the *SLC26A4* variant c.919–2A>G (p.?) in both parents.

Following genetic counseling, Couples 12 and 185 elected to undergo preimplantation genetic testing (PGT) and awaited embryo implantation following confirmation of disease carrier status. Couple 55 opted for natural conception and proceeded with prenatal diagnosis via amniocentesis, which revealed that the fetus was an *SMN1* carrier. Based on this result, they chose to continue the pregnancy.

The remaining eight ARCs were identified based on missense variants in the *G6PD* gene, an X-linked condition in which female carriers typically remain asymptomatic. Among these cases, one female participant carried a homozygous *G6PD* variant, while the others were heterozygous carriers with missense variants.

## 4 Discussion

In China, carrier screening is increasingly recognized as a valuable tool for assessing the risk of birth defects during the preconception period or the first trimester of pregnancy. This technology is gaining acceptance among individuals of childbearing age. In 2024, several expert consensus documents were released in China outlining the clinical application of carrier screening. These guidelines defined the scope of carrier screening, clarified the target population and principles for disease inclusion, and emphasized its role as a preventive measure aimed at addressing the root causes of birth defects (Liu, 2024; Huang and Xu, 2024; Lu et al., 2024). One of these consensus statements highlighted the necessity of tailoring carrier screening programs to the specific characteristics of different regions in China (Lu et al., 2024). Consequently, compiling regional clinical data on carrier screening is essential for identifying common disease-causing genes and developing more cost-effective screening strategies and technologies.

When evaluating the implementation of regional ECS strategies to prevent severe single-gene recessive diseases in offspring, it is crucial to ensure that the selected diseases and genes accurately reflect the most prevalent AR conditions in the local population. Although the sample size in this study was relatively limited (n = 440), the findings identified 75 conditions with a carrier frequency of at least 1/200. Elevated carrier rates were observed for AR conditions such as non-syndromic hearing loss, alpha-thalassemia, Crigler-Najjar syndrome, Wilson’s disease, and glucose-6-phosphate dehydrogenase deficiency within Fujian Province. The focus on locally prevalent pathogenic variants provides valuable insights and underscores the relevance of this research. Moreover, this study serves as a model for pilot screening initiatives and offers a framework for establishing carrier screening programs in other geographic regions.

It is important to emphasize that carrier screening does not diagnose disease in an individual but rather identifies the risk of having offspring affected by a genetic disorder.

### 4.1 Performance of the designed ECS panel

The ECS panel developed for this study targeted 332 genes, offering a cost-effective alternative to virtual panels based on whole exome sequencing (WES). Given economic considerations, this approach is more suitable for large-scale implementation. Unlike WES, which sequences the entire exome, the targeted panel focuses on a smaller subset of genes, allowing for greater sequencing depth. This enhanced coverage improves the accuracy of CNV detection and minimizes misalignment errors. The methodology employed in this study utilized a 332-gene panel to detect SNVs, indels, and CNVs, with specialized precision for complex genomic regions such as *CYP21A2*, *HBA1*/*HBA2*, and *SMN1*. This approach proved effective in identifying relatively high-incidence recessive conditions in the Chinese population.

A total of 511 variants were detected, involving 133 genes and 123 recessive genetic disorders, with an average pathogenic variant burden of 1.16. The overall carrier rate was 69.09%, while the percentage of ARC was 6.36% (or 2.73% when excluding the effect of *G6PD* variants).

Recent international studies using panels containing 176–283 genes have reported carrier rates ranging from 32% to 71.85%, with the proportion of ARC ranging from 1.6% to 9.46% (Marinakis et al., 2024; Strauss et al., 2023). A large-scale study in the Chinese population demonstrated a carrier rate of 62.33% and the percentage of ARC was 2.12% (Huang et al., 2024). These findings are consistent with the results of the present study.

Within the GS group, 64.56% of individuals carried at least one pathogenic variant, whereas the carrier rate increased to 71.63% in the MAR group (Table 1). A previous study by Xi et al. reported that 46.73% of couples undergoing ART were carriers of PLPVs using a 201-gene panel (Xi et al., 2020). Notably, other panels designed for the Chinese population, such as those developed by Guo and Haque, have demonstrated higher diagnostic yields (Guo and Gregg, 2019; Haque et al., 2016).

In this study, 6.36% (n = 14) of tested couples were identified as ARCs for conditions such as non-syndromic hearing loss and *G6PD* deficiency (with a 1/2 risk for X-linked recessive diseases or a 1/4 risk for autosomal recessive conditions). Among these couples, 2.73% (n = 6) carried AR variants affecting the same gene, a rate that aligns with previous estimates suggesting that the ARC proportion in the Chinese population ranges between 2.26% and 3.2% (Xi et al., 2020; Shi et al., 2021). Given that China has an estimated 9 to 10 million newborns annually, our analysis demonstrates th ARC profiles of autosomal/X-link recessive carrier (Marinakis et al., 2024): (1) The percentage of ARC for X-linked recessive disorders was 3.64%, identifying 350,000 couples at risk of transmitting X-linked disorders; (2) Analysis of isolated autosomal recessive conditions demonstrated an ARC percentage of 2.73%, corresponding to 250,000 couples with elevated genetic risk.

Further subgroup analysis indicated that the percentage of ARCs in the GS group was 6.33% (5/79), whereas the percentage of ARCs with a history of adverse pregnancy outcomes was 6.38% (9/141). A chi-squared test conducted using SPSS demonstrated no statistically significant difference between these two groups (χ^2^ = 0.00, *p* = 0.987). Three couples in the MAR group whose offspring had genetic conditions were identified; however, none carried additional variants within the 332-gene panel. Following diagnosis, four couples pursued PGT-assisted pregnancy services. Additionally, in the GS group, two couples were identified as carriers for Krabbe disease (*GALC*, MIM: #245200) and phenylketonuria (*PAH*, MIM: #261600), respectively, with both couples previously unaware of their shared pathogenic variants.

These findings underscore the importance of integrating ethnicity-based ECS into routine prenatal care and MAR programs to prevent birth defects, further reinforcing the clinical utility of ECS in reproductive health and public health initiatives.

### 4.2 Characteristics of detected variant genes and diseases

A precise method was developed for analyzing gene variations within highly homologous regions, specifically *CYP21A1P*/*CYP21A2*, *HBA1*/*HBA2*, and *SMN1*/*SMN2*. This approach significantly improved diagnostic accuracy while reducing the time and costs associated with individual gene testing. Among the 511 variants detected, 91.59% were SNVs or indels, while 8.41% were exon-level CNVs. The incorporation of exon-level CNV detection substantially increased the positive detection rate, with copy number analysis of these genes yielding higher detection rates than those observed in the 200 least common genes in this study, consistent with previous findings (Hogan et al., 2018). These results underscore the importance of incorporating CNV exon detection into future ECS panel designs in China. However, most carrier screening programs in China do not currently utilize NGS as a primary tool for exon-level CNV detection (Zhang et al., 2020; Chen et al., 2023; Xi et al., 2020; Guo and Gregg, 2019; Sherman et al., 2022; Haque et al., 2016; Shi et al., 2021; Hogan et al., 2018; Chau et al., 2022).

Analysis of gene misalignments in highly homologous genomic regions identified 9 individuals within this cohort as carriers of *CYP21A2* variants, corresponding to a carrier rate of 2.05% for 21-hydroxylase deficiency (21-OHD). The *CYP21A2* gene has a pseudogene, *CYP21A1P*, with 95% sequence homology, and gene conversion events between these loci are responsible for approximately 90% of 21-OHD cases (Cavalli and Heard, 2019). Traditional paired-end reads from Illumina sequencing often result in misidentifications of variants in these regions, leading to false positives or false negatives. Additionally, recombination between *CYP21A2* and *CYP21A1P* can introduce CNV errors due to read mismatches. Using the proposed method, misalignment rates within these genes were analyzed, enabling the accurate identification of variants in *CYP21A2* (Lee et al., 2021). All detected variants were validated using MLPA or Sanger sequencing.

It is well-established that the intronic splicing variants of *CYP21A2* such as c.293–13C/A>G (p.?) and c.-126C>T (p.?) can lead to the complete loss of enzyme activity due to recombination events between *CYP21A2* and *CYP21A1P*, resulting in the classic salt-wasting (SW) phenotype. The V282L variant, detected in one sample in exon 7, is associated with the non-classic phenotype, which retains approximately 30%–50% residual enzyme activity (Wang et al., 2020; Umino et al., 2019). Additionally, three common pathogenic variants—c.518T > A (p.Ile173Asn), c.955C>T (p.Gln319*), and *CYP21A2* exon 1–10 deletions—were identified, all of which are linked to simple virilizing and SW phenotypes of 21-OHD (Simonetti et al., 2018).

Given the high incidence and clinical severity of 21-OHD in China, developing of an NGS-based method capable of detecting a broad spectrum of *CYP21A2* pathogenic variants, including large deletions, could significantly enhance the clinical utility of ECS (Wang et al., 2020; Umino et al., 2019; Simonetti et al., 2018).

Among the 12 identified disease categories, metabolic/endocrine anomalies exhibited the highest prevalence of genes with frequent variants and PLPVs. A total of 59 genes were implicated, constituting 44% (59/133) of all genes with identified variants. This category also contained the highest concentration of genes with carrier rates exceeding 1/100, totaling 10 genes (Supplementary Figure S1). The uneven distribution of variants across disease categories is an important consideration when designing and optimizing ECS panels.

Among the 10 most frequently observed genes, variants in *GALT* and *GAA* were not previously identified in hotspot regions (see Supplementary Table S4). Additionally, three individuals (one female and two males) were identified as carriers of the *CFTR* c.4056G>C (p.Gln1352His) variant, which is commonly associated with congenital absence of the vas deferens in Chinese individuals (Luo et al., 2021). One of these three participants reported fertility issues, further supporting the pathogenic significance of this variant.

Neonatal hyperbilirubinemia, which is often linked to *UGT1A1* and *G6PD* variants, is highly prevalent in China, affecting approximately 34.4% of full-term neonates and is generally considered treatable (Yang et al., 2016). *G6PD* deficiency is one of the most frequently occurring X-linked enzymopathies in Chinese populations, particularly in Guangdong and Guangxi provinces (Luzzatto et al., 2016). In addition to their well-established role in hemolytic anemia, *G6PD* variants have been associated with recurrent miscarriages, leading recent studies to propose their inclusion in carrier screening programs (Huang et al., 2019; Puleston et al., 2017). Given the high carrier rate in Fujian Province, *G6PD* was included in this study to further investigate its potential association with fertility-related complications. In the Han Chinese population, the primary *G6PD* variants are c.1388G>A and c.1376G>T, with significant differences in enzyme activity observed between homozygous females (*p* = 0.020) (Lin et al., 2018; He et al., 2020). The carrier frequency observed in this cohort is consistent with previous reports and suggests a possible association with adverse obstetric history.

Auditory and hematologic disorders were also frequently observed in this cohort (Supplementary Table S4). Within these disease categories, fewer than half of the associated genes accounted for more than 90% of the CCR. The *GJB2* c.109G > A variant was detected 78 times, a finding consistent with its known association with mild and late-onset hearing loss in Asian populations (Ogawa et al., 2007). Although prenatal diagnosis is not currently recommended for potential homozygous carriers of c.109G>A, studies indicate that when this variant occurs in combination with other *GJB2* variants, it may lead to severe early-onset hearing loss (Chau et al., 2022; Tian et al., 2022; Yang et al., 2021). Consequently, the ECS panel in this study was designed to include detection and reporting of this variant.

Regarding hematologic disorders, three blood-related conditions are commonly addressed in ECS studies, with alpha-thalassemia being one of the most extensively studied in Fujian Province (Pan et al., 2022). The alpha-thalassemia detection rate in this cohort was 28.24%, with the most frequent genotype being αα/--^SEA^, observed in 64.80% of cases. Other genotypes, such as αα/--^THAI^ (0.41%) and HKαα/--^SEA^ (0.03%), were also identified. Among all *HBA1*/*HBA2* variants, the most frequently observed carrier genotype was αα/--^SEA^ (60.87%), followed by αα/-α^3.7^ (30.43%) and αα/-α^4.2^ (8.70%).

Additionally, pathogenic *HBB* variants associated with hepatitis B virus infection, specifically c.126_129del (p.Phe42Leufs*19) and c.316–197C>T (p.?), were detected in four and three individuals, respectively. These variants are among the most common *HBB* variations observed in southern China (Chau et al., 2022).

Nervous system anomalies ranked third in terms of CCR in this analysis (Supplementary Table S9). One example is the *SKIC* gene, located at 5q15 on the long arm of chromosome 5, where biallelic pathogenic variants are associated with tricho-hepato-enteric syndrome 1 (OMIM#614589) (Bourgeois et al., 2018). The carrier frequency of *SKIC* in Fujian province appears relatively elevated compared to reported rates in North and Central China, with an AF of 0.91% observed in this study. Baxter et al. previously reported destructive germline variants in the *SKIC3* gene in 48 cases (4.17%) of FOXP3-negative IPEX-like disease, suggesting that similar variants may exhibit regional variability in disease prevalence (Baxter et al., 2022). These findings imply that the carrier spectrum and disease burden for nervous system-related genetic conditions may vary across geographic populations.

In this study, dermatologic, hepatic, and respiratory anomalies did not include any genes with a carrier rate exceeding 1/100, suggesting that screening for diseases within these categories may have limited cost-effectiveness for the Fujian population. This finding indicates that the absence or rarity of pathogenic variants in these systems may not justify their inclusion in future regional carrier screening panels.

Additionally, some commonly observed variants in this study differed from those reported in previous studies. Xi et al. reported a 1.4% carrier rate for *CYP1B1* in Chinese individuals undergoing ART, with the c.319C > G (p.Leu107Val) variant representing 85% of detected *CYP1B1* variants (Xi et al., 2020). In contrast, in this study, five pathogenic *CYP1B1* variants were identified, resulting in a carrier rate of 1.1%. Furthermore, while prior Chinese studies have attributed 19.3% of familial Mediterranean fever cases to variants in the *MEFV* gene—predominantly the E148Q variant (Wu et al., 2018)—in this study, five cases with potentially pathogenic *MEFV* variants were detected, all of which were c.2282G > A (p.Arg761His), resulting in a 1.1% carrier rate.

## 5 Conclusion

The findings of this study indicate that NGS-based ECS is a promising approach for genetic testing in pregnant couples and those seeking MAR, particularly in the identification of ARCs to prevent severe recessive monogenic diseases in offspring. Expanding access to these services in clinical settings may enhance equitable reproductive care. Birth defects associated with genetic factors may be largely preventable through ECS programs that target region-specific recessive conditions.

The identification of recurrently altered genes and their associated PLPVs provides valuable insights into the genetic disease spectrum in the Fujian population. Notably, tricho-hepato-enteric syndrome 1 attributed to *SKIC3* variants, exhibits a higher carrier rate in this population, while *CYP1B1*-related glaucoma presents with unique hotspot variants. These findings highlight important directions for genetic disease research and underscore the need for cost-effective ECS panels tailored to regional populations. Additionally, this study demonstrates that enhanced NGS and bioinformatics approaches, including pseudogene integration and exon-level CNV analysis, can improve the diagnostic yield of ECS by approximately 11.36% (50/440), representing a key advancement in carrier screening methodology.

## 6 Limitations

Several limitations should be considered when interpreting these findings. ECS has inherent constraints that may result in false-negative results, in part due to challenges in variant interpretation. Pathogenicity was assessed following ACMG guidelines, supplemented by available evidence at the time of analysis. Future revisions to these guidelines or the emergence of new evidence may lead to reclassification of certain variants, which could affect reported carrier frequencies and the percentage of ARCs.

Additionally, the annotation of genetic diseases in this study was based on public databases, which may introduce the potential for false negatives if rare or ethnicity-specific gene variants were absent from these resources (Cook et al., 2024; Boonsawat et al., 2022). Furthermore, some variants included in this study may be associated with milder clinical presentations, limiting their immediate clinical significance. Finally, the study cohort from Fujian Province may not fully capture the broader genetic diversity of subpopulations across China, and additional studies in larger and more diverse cohorts are warranted to validate these findings.

## Data Availability

The data presented in the study are deposited in the dbSNP repository, accession link https://www.ncbi.nlm.nih.gov/SNP/snp_viewBatch.cgi?sbid=1063651.

## Abbreviations

NGS, Next-generation sequencing; ECS, Expanded carrier screening; SNVs, Single nucleotide variants; CNVs, Copy number variations; PLPVs, Pathogenic or likely pathogenic variants; ARCs, At-risk couples; GS group, Genetic screening group; MAR group, Medically assisted reproduction group; MAR-group A, Infertility group; MAR-group B, Spontaneous abortion; MAR-group C, Second and third trimesters ultrasound or postnatal abnormalities; CCR, Cumulative carrier rate; MLPA, Multiplex ligation-dependent probe amplification; CNV-exons, Exons with copy number variations; AR, Autosomal recessive; AF, Allele frequency; SMA, Spinal muscular atrophy; 21-OHD, 21- hydroxylase deficiency; SW, Salt-wasting; SV, Simple virilizing; FMF, Familial Mediterranean fever.
