# Supplementary material for: Regional patterns of genetic variants in expanded carrier screening: a next-generation sequencing pilot study in Fujian Province, China
Source: Front Genet. 2025 May 12;16:1527228. doi: 10.3389/fgene.2025.1527228 (PMC12104066; doi:10.3389/fgene.2025.1527228)
Supplement: Supplementary file 2 [file DataSheet1.docx]

**Regional Variant Patterns in Expanded Carrier Screening Using Next-Generation Sequencing: A Pilot Study in Fujian Province, Southeast China**

# Criteria of disease selection of ECS panel

Several aspects were deliberated to determine the selection of conditions for ECS panel. The panel was designed to detect genes containing misalignment issues related to homology, including SMN1, HBA1/HBA2, GBA, CYP21A2, CYP21B1, VWF, DCRE1C, ALMS1, and IDS, according to the method described in our previous study [[1]](#L1). Firstly, conditions were satisfying the guidelines/recommendations of ACMG, ACOG, NSGC, PQF, and SMFM [[2]](#L2), as well as having a carrier frequency (CF) >1/500 in any ethnicity [[3]](#L3). Secondly, we included severe childhood‐onset disorders with highly penetrant phenotypes, high‐prevalence monogenic diseases with moderate phenotypes, and disabilities that impact the quality of life for the entirety of the patient's life, such as severe hearing loss and blindness [[4]](#L4). Finally, the selected genes must be detected with NGS with a defined native detection rate and variants could be validated by methods like Sanger, MLPA, and qPCR.

In summary, the 332 genes of the 332-ECS panel involved the disease systems （Table S1）: metabolic/endocrine (41.7%), nervous (16.5%), musculoskeletal (12.0%), and then followed by hematological, ophthalmologic, skin, immunity, auditory, kidney, multi-system, others, respiration and liver.

The mode of inheritance of these genes was AR (n=303, 91.3%), followed by X-linked (n=27, 8.1%), and finally AR/Digenic (n=2, 0.6%). The severity of the disease was evaluated according the to method developed by Arjunan et al [[5]](#L5). 16 diseases have been evaluated as “MILD”, and the rest of the diseases are classified as a severity more than the level of “Moderate”.

The designated target regions included exon regions and 20-bp flanking regions of selected ECS genes, which were annotated from NCBI RefSeq, and P/LP variants with ClinVar star 2+ (i.e., multiple submitters with assertion criteria, expert panel, or practice guideline without conflicting interpretations) of intron, intergenic and UTR regions were also included in our panel.

# Genomic Sequencing and Data Analysis

Peripheral blood samples from 440 participants were drawn with informed consent and genomic DNA (gDNA) were extracted using the QIAamp® DNA Blood Mini kit following the manufacturer’s protocol (Qiagen, Germantown, MD, USA). All gDNA (300-500 ng) was sheared, size selected (400–600 bp), ligated to sequencing adapters, and PCR amplified following standard library preparation. The post-PCR library was then used for exome capture using the 332-gene panel and synthesized by Integrated DNA Technologies, Coralville, IA, USA. Exome-enriched samples were sequenced (2×150 bp) on an Illumina Novaseq (Illumina, Inc., San Diego, CA, USA). Raw image files were processed using bcl2fastq2 conversion software v2.20 (Illumina, Inc., San Diego, CA, USA).

The sequencing reads were aligned to the human reference genome (hg19/GRCh37) using the Burrows–Wheeler alignment tool, and PCR duplicates were removed using Picard software v1.57 (http://picard.sourceforge.net/). The genomic analysis toolkit GATK4 (https://software.broadinstitute.org/gatk/) was employed for variant discovery. Variant annotation and interpretation were conducted using the ANNOVAR software (http://www.openbioinformatics.org/annovar/).

A newly developed method was used to identify genes containing misalignment issues related to homology, including *SMN1*, *HBA1/HBA2*, *GBA*, *CYP21A2*, *CYP21B1*, *VWF*, *DCRE1C*, *ALMS1*, and *IDS* [[1]](#L6). The internally developed software CNV-exon, a coverage-based CNV detection tool [[6]](#L7), was used to analyze CNVs, especially for exon-level heterozygous deletions or amplifications.

# Variant Interpretations

The variants interpretation was utilized a protocol descriped by Zhang et al.[7] Variant frequencies were determined in the 1000 Genomes Project, Exome Variant Server (http://evs.gs.washington.edu/EVS), ExAC (http://exac.broadinstitute.org/), and gnomAD v2.1.1 (http://gnomad-old.broadinstitute.org/) and in-house database ISoGenetics to remove common SNPs (minor allele frequency > 0.5%). Then, nonsynonymous, splicing and frameshift, non-frameshift variants, as well as variants located in splice sites within 10 base pairs of an exon, were prioritized for study. The pathogenicity of missense variants were predicted by using REVEL(https://sites.google.com/site/revelgenomics/), as well as SIFT (http://sift.jcvi.org), PolyPhen-2 (http://genetics.bwh.harvard.edu/pph2), MutationTaster (http://www.mutationtaster.org), and CADD (http://cadd.gs.washington.edu). SpliceAI ( https://github.com/Illumina/SpliceAI ) were used for evaluating the effects on splicing. OMIM, Clinvar, and HGMD was used to identify variants reported as pathogenic in published studies. Pathogenicity of variants was determined following the current ACMG guidelines.All the identified variants were classified into five categories: “pathogenic,” “likely pathogenic,” “uncertain significance,” “likely benign,” and “benign” according to the ACMG guidelines for the interpretation of genetic variants [8]. All the single-nucleotide variants (SNVs) and copy number variants (CNVs) classified as “pathogenic” or “likely pathogenic” (P/LP) were confirmed through Sanger sequencing and MLPA.

# Sanger Sequencing

Sequencing was performed in both forward and reverse directions using 1.6 µl cleanup PCR products, 0.8 µl BigDye, and 2.0 µl M13 sequencing primers (1.6 µM) in each reaction.

1. **Multiplex Ligation-dependent Probe Amplification**

MLPA was performed with 100 ng genomic DNA per reaction using the SALSA MLPA probe mixes P050-C1 to analyze *CYP21A2*, P060 to analyze *SMN1*, P140 to analyze *HBA1/HBA2*, P209 to analyze *GLDC*, P387 to analyze *NPHP1*, P368 to *DCLRE1C*, P031/032 to analyze *FANCA*, P441 to analyze *SACS*, P116 to analyze *SGCG*, and P321/322 to analyze *VPS13B*, according to the manufacturer’s recommendations (MRC Holland, Amsterdam, The Netherlands). Quality control and data analysis were conducted using the Coffalyser.net software (MRC Holland, [www.mlpa.com](http://www.mlpa.com/)).

1. **Quantitative real-time PCR**

The CNVs of *PMM2* and *FAM161A* were measured by quantitative real-time PCR (RT-qPCR) using SuperReal PreMix Plus (SYBR Green) (TIANGEN, China) in an QuantStudi™ 6 Flex Real-Time PCR System (Thermo Fisher Scientific, US), ALB served as the internal reference, and data were analyzed with the 2^-△△Ct^ method. The qPCR mixture contained 5ul 2×SuperReal PreMix Plus, 0.3ul Forward Primer (10uM), 0.3ul Reverse Primer (10uM), 10ng DNA template, 0.2ul 50×ROX Reference Dye, and up to 10ul using RNase-Free Water. qPCR conditions were as follows: 2min at 50℃, 10min at 95℃, followed by 40 cycles of 95℃ for 15s and 60℃ for 1min, 15s at 95℃, 1min at 60℃, 30s at 95℃, 15s at 60℃. Gene specific primers for qPCR analysis was designed using the NCBI Nucleotide Search database (https://www.ncbi.nlm.nih.gov/nuccore/) and NCBI Primer-BLAST Tool (https://www.ncbi.nlm.nih.gov/tools/primer-blast/).

**References**

1. Lee CCY, Yen H, Zhong AW, Gao H. Resolving misalignment interference for NGS-

based clinical diagnostics. Hum Genet. 2021;140:477-92.

1. Edwards JG, Feldman G, Goldberg J, Gregg AR, Norton ME, Rose NC, et al. Expanded Carrier Screening in Reproductive Medicine-Points to Consider. Obstet Gynecol. 2015;125:653-62.
2. Ben-Shachar R, Svenson A, Goldberg JD, Muzzey D. A data-driven evaluation of the

size and content of expanded carrier screening panels. Genet Med. 2019;21:1931-9.

1. Lazarin GA, Hawthorne F, Collins NS, Platt EA, Evans EA, Haque IS. Systematic Classification of Disease Severity for Evaluation of Expanded Carrier Screening Panels. PLoS ONE. 2014;9:e114391.
2. Arjunan A, Bellerose H, Torres R, Ben-Shachar R, Hoffman JD, Angle B, et al. Evaluation and classification of severity for 176 genes on an expanded carrier screening panel. Prenat Diagn. 2020;40:1246-57.
3. Strom SP, Hossain WA, Grigorian M, Li M, Fierro J, Scaringe W, et al. A Streamlined

Approach to Prader-Willi and Angelman Syndrome Molecular Diagnostics. Front Genet. 2021;12:608889.

1. Zhang J, Yao Y, He H, Shen J. Clinical Interpretation of Sequence Variants. Curr Protoc Hum Genet. 2020;106(1):e98.
2. Richards S, Aziz N, Bale S, Bick D, Das S, Gastier-Foster J, et al. Standards and guidelines for the interpretation of sequence variants: a joint consensus recommendation of the American College of Medical Genetics and Genomics and the Association for Molecular Pathology. Genet Med. 2015;17:405-24.
